# Diffusion‐tensor magnetic resonance imaging captures increased skeletal muscle fibre diameters in Becker muscular dystrophy

**DOI:** 10.1002/jcsm.13242

**Published:** 2023-05-01

**Authors:** Donnie Cameron, Tooba Abbassi‐Daloii, Laura G.M. Heezen, Nienke M. van de Velde, Zaïda Koeks, Thom T.J. Veeger, Melissa T. Hooijmans, Salma el Abdellaoui, Sjoerd G. van Duinen, Jan J.G.M. Verschuuren, Maaike van Putten, Annemieke Aartsma‐Rus, Vered Raz, Pietro Spitali, Erik H. Niks, Hermien E. Kan

**Affiliations:** ^1^ C.J. Gorter MRI Center, Department of Radiology Leiden University Medical Center Leiden The Netherlands; ^2^ Department of Human Genetics Leiden University Medical Center Leiden The Netherlands; ^3^ Department of Neurology Leiden University Medical Center Leiden The Netherlands; ^4^ Duchenne Center Netherlands Leiden The Netherlands; ^5^ Radiology and Nuclear Medicine Amsterdam University Medical Center, University of Amsterdam, Amsterdam Movement Sciences Amsterdam The Netherlands; ^6^ Department of Pathology Leiden University Medical Center Leiden The Netherlands

**Keywords:** Becker muscular dystrophy, diffusion‐tensor MRI, histopathology, immunohistochemistry, skeletal muscle

## Abstract

**Background:**

Becker muscular dystrophy (BMD) is an X‐linked disorder characterized by slow, progressive muscle damage and muscle weakness. Hallmarks include fibre‐size variation and replacement of skeletal muscle with fibrous and adipose tissues, after repeated cycles of regeneration. Muscle histology can detect these features, but the required biopsies are invasive, are difficult to repeat and capture only small muscle volumes. Diffusion‐tensor magnetic resonance imaging (DT‐MRI) is a potential non‐invasive alternative that can calculate muscle fibre diameters when applied with the novel random permeable barrier model (RPBM). In this study, we assessed muscle fibre diameters using DT‐MRI in BMD patients and healthy controls and compared these with histology.

**Methods:**

We included 13 BMD patients and 9 age‐matched controls, who underwent water‐fat MRI and DT‐MRI at multiple diffusion times, allowing RPBM parameter estimation in the lower leg muscles. Tibialis anterior muscle biopsies were taken from the contralateral leg in 6 BMD patients who underwent DT‐MRI and from an additional 32 BMD patients and 15 healthy controls. Laminin and Sirius‐red stainings were performed to evaluate muscle fibre morphology and fibrosis. Twelve ambulant patients from the MRI cohort underwent the North Star ambulatory assessment, and 6‐min walk, rise‐from‐floor and 10‐m run/walk functional tests.

**Results:**

RPBM fibre diameter was significantly larger in BMD patients (*P* = 0.015): mean (SD) = 68.0 (25.3) μm versus 59.4 (19.2) μm in controls. Inter‐muscle differences were also observed (*P* ≤ 0.002). Both inter‐ and intra‐individual RPBM fibre diameter variability were similar between groups. Laminin staining agreed with the RPBM, showing larger median fibre diameters in patients than in controls: 72.5 (7.9) versus 63.2 (6.9) μm, *P* = 0.006. However, despite showing similar inter‐individual variation, patients showed more intra‐individual fibre diameter variability than controls—mean variance (SD) = 34.2 (7.9) versus 21.4 (6.9) μm, *P* < 0.001—and larger fibrosis areas: median (interquartile range) = 21.7 (5.6)% versus 14.9 (3.4)%, *P* < 0.001. Despite good overall agreement of RPBM and laminin fibre diameters, they were not associated in patients who underwent DT‐MRI and muscle biopsy, perhaps due to lack of colocalization of DT‐MRI with biopsy samples.

**Conclusions:**

DT‐MRI RPBM metrics agree with histology and can quantify changes in muscle fibre size that are associated with regeneration without the need for biopsies. They therefore show promise as imaging biomarkers for muscular dystrophies.

## Introduction

Becker muscular dystrophy (BMD) is an X‐linked disorder caused by pathogenic variants in the *DMD* gene that allow production of internally deleted dystrophin proteins with reduced functionality. It occurs in approximately 1.5–6 male births in every hundred thousand and is characterized by slow, progressive muscle damage, concomitant muscle weakness and substantial clinical variability. Hallmarks of BMD include membrane instability in muscle fibres, variability in fibre size and replacement of skeletal muscle tissue with fat and fibrosis, which follows a specific pattern of muscle involvement.^1^ To date, there are no treatments that significantly curb progression of the disease, but several pharmaceuticals are in development (www.clinicaltrials.gov: NCT03238235, NCT05160415 and NCT03236662). In order to test these interventions in clinical trials, sensitive and specific outcome measures are needed.

Skeletal muscle histopathological changes are central to BMD. The lack of fully functional dystrophin is associated with sarcolemma instability and vulnerability to injury, which leads to fibre necrosis, inflammation and regeneration. Due to chronic inflammation and tissue remodelling, regeneration is impaired,^2^ which is evident from histopathological features such as fibre‐size variation, central nuclei and regenerating fibres.^3^ The end stage of repeated cycles of damage and repair is replacement of muscle fibres with fat and connective tissue, at which point muscle function is irrevocably lost. Histological assays can capture many of the aforementioned features. Indeed, they have been used as endpoints in an uncontrolled study^4^ and a phase II clinical trial^5^ in BMD. However, biopsies are invasive, are difficult to repeat and only capture a local, perhaps non‐representative sample of a single muscle, limiting their generalizability to other muscles.

Quantitative magnetic resonance imaging (MRI) offers large‐volume, multi‐muscle data that have already proved invaluable in understanding disease progression in muscular dystrophies. Chemical‐shift‐based fat‐water techniques are most frequently used, offering measures of fat replacement in skeletal muscle.^6^, ^7^ However, fat replacement is considered irreversible and represents an end stage of muscle pathology. Diffusion‐tensor MRI (DT‐MRI) offers a potential non‐invasive means of assessing early pathological changes. It is sensitive to the Brownian motion of water molecules, and it can quantify the internal microstructure and architecture of muscle tissue by measuring patterns of water diffusion therein. Previous applications of DT‐MRI in BMD and Duchenne muscular dystrophy (DMD) showed no, or inconsistent, differences between patients and controls.^8^, ^9^ However, these studies used conventional DT‐MRI, which—with its short diffusion times—probes a scale of only ~20 μm, and so cannot fully explore muscle's hierarchy of myofibrils and myofibres (~1–100 μm). Recent, advanced applications of DT‐MRI are capable of longer diffusion times, thereby exploring a broader scale of muscle structures. These have been shown to be sensitive to muscle damage in DMD.^10^ Obtaining multiple diffusion times permits use of the random permeable barrier model (RPBM),^11^, ^12^, ^13^, ^14^ which represents muscle as a network of randomly oriented, semi‐permeable membranes, resembling histology in cross‐section. The RPBM provides non‐invasive measures of muscle biophysical parameters—fibre diameter and membrane permeability—as such appearing ideal for capturing the heterogeneous fibre sizes and increased sarcolemma membrane permeability demonstrated in BMD,^3^, ^15^ as a promising alternative to muscle biopsies. Indeed, recent preclinical work using the RPBM has shown differences in skeletal muscle microstructure between *mdx* mice and controls, along with alterations throughout the course of the disease.^16^ What remains is to apply the RPBM in patients, as muscle pathology in the *mdx* mouse is not representative of human pathology.

In this exploratory study, we apply DT‐MRI and the RPBM in the lower leg muscles of BMD patients and healthy controls, comparing parameters, including muscle fibre size and permeability, between the two groups. We then compare these measures to histologically assessed fibre size and fibrosis in the tibialis anterior (TA) muscle. These analyses may identify DT‐MRI as an imaging biomarker for muscular dystrophies that can capture regeneration‐associated changes in muscle fibre size without the need for biopsies.

## Methods

### Participant inclusion and muscle biopsies

MRI scans were run from 2017 to 2018 as part of a BMD natural history study that commenced in 2014.^17^ We included 13 BMD patients, with a mean [range] age of 41 [20–59] years, from the Dutch Dystrophinopathy Database, and 9 healthy, male controls, with a mean [range] age of 44 [23–65] years, from the Leiden University Medical Center Radiology database. BMD diagnoses were genetically confirmed (pathogenic variants in the *DMD* gene). Of the 13 patients, 1 was wheelchair‐bound and 12 were ambulant.

Six BMD patients underwent both DT‐MRI and muscle biopsy procedures, and we included six additional biopsy samples from patients who did not undergo DT‐MRI. A further 26 biopsies were included from an earlier BMD natural history study, run in 2011,^18^ yielding 38 BMD biopsy samples in total. Finally, 15 samples from male controls (mean [range] age = 40 [23–59] years) were identified from a local Department of Pathology database of patients who underwent biopsies for musculoskeletal complaints. These samples were selected on the basis that no neuromuscular disease had been diagnosed. Biopsies were performed as described previously.^19^ In short, samples were collected under local anaesthesia from the TA muscle of the right leg—the contralateral leg relative to the MRI scans. A 1.5‐ to 2‐cm incision was made in the skin and fascia, and three to four muscle samples were collected using a conchotome. These were then snap‐frozen in liquid‐nitrogen‐cooled isopentane and stored at −80°C prior to cryosectioning.

Ethical approval was obtained from the local research ethics committee (Protocols P10.133, P12.214 and P14.243), and participants gave written informed consent after receiving a detailed study description, in accordance with the Declaration of Helsinki.

### Magnetic resonance imaging

Scans were performed using a 3‐tesla Ingenia wide‐bore system (Philips, Best, the Netherlands) with a 16‐element anterior torso array and 12‐element built‐in posterior array for signal reception. Participants were positioned supine, feet‐first with their feet in a neutral position and their ankles supported by sandbags to avoid gross motion. To enable RPBM analyses, we applied short‐diffusion‐time spin‐echo DT‐MRI and long‐diffusion‐time stimulated‐echo DT‐MRI, with the latter being acquired at mixing times of 100 and 300 ms. Also included in the protocol were chemical‐shift‐based water‐fat separation scans acquired using the Dixon technique. Further MRI details are given in the Supporting Information, *Data* *S1*.

#### Diffusion‐tensor magnetic resonance imaging processing pipeline

**Figure 1:**
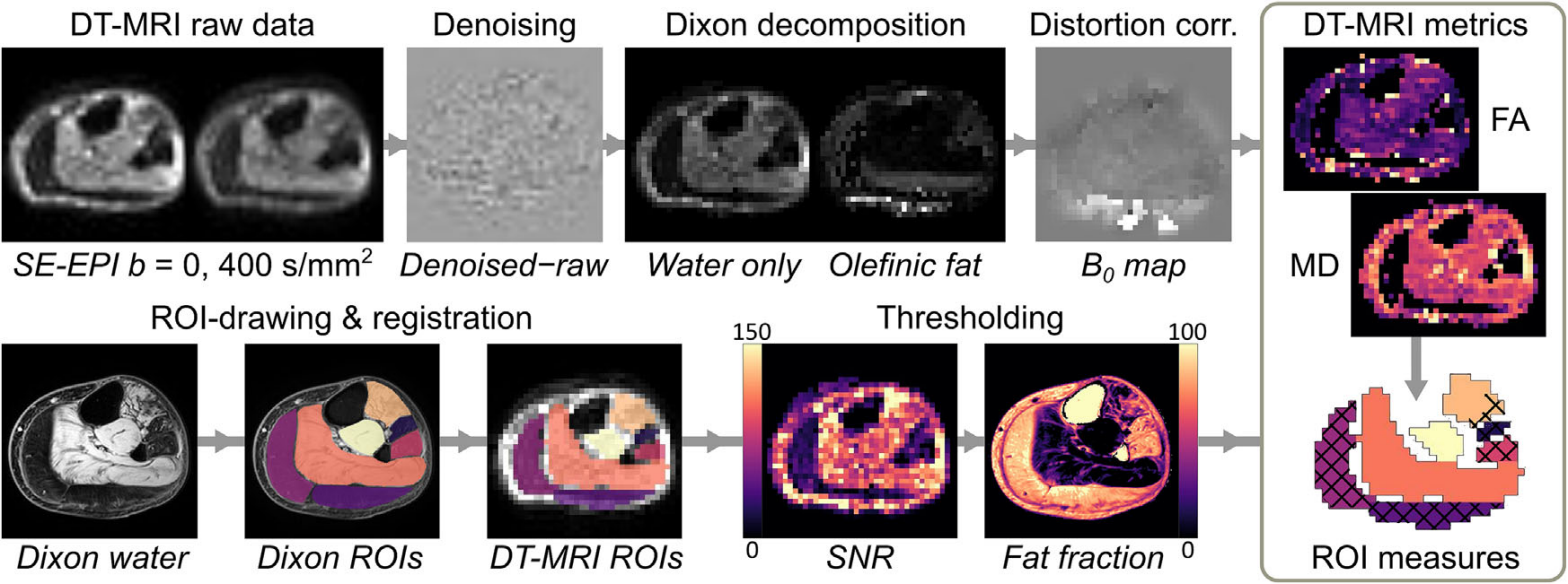
Schematic showing the diffusion‐tensor magnetic resonance imaging (DT‐MRI) processing pipeline for this study. Representative axial spin‐echo echo planar imaging (SE‐EPI) DT‐MRI and chemical‐shift‐based water‐fat separation, or ‘Dixon’s;, images from the lower leg are shown for a 59‐year‐old Becker muscular dystrophy patient. The top row shows the DT‐MRI raw data, which undergo denoising, Dixon fat‐water decomposition to remove olefinic fat, and distortion correction. The bottom row shows region of interest (ROI) drawing on Dixon images, registration of ROIs to the DT‐MRI data and pixel‐wise thresholding based on low signal‐to‐noise ratio (SNR) and high fat fraction. ROIs, with excluded pixels indicated by a crosshatch pattern, are then used to obtain conventional DT‐MRI and random permeable barrier model metrics per muscle. FA, fractional anisotropy; MD, mean diffusivity.

Data were processed using an in‐house MATLAB‐based pipeline (MATLAB 2019a, The MathWorks, Natick, CA, USA), illustrated in *Figure* *1*, ^20^ and detailed in *Data* *S1*. The code is available for download at https://git.lumc.nl/neuroscience/standardiseddtipipeline.

#### Diffusion‐tensor magnetic resonance imaging region‐of‐interest analyses

Regions of interest (ROIs) were drawn on Dixon water images using MIPAV (v8, https://mipav.cit.nih.gov) in the TA, soleus (SOL), medial gastrocnemius (GCM), lateral gastrocnemius (GCL), tibialis posterior (TP), peroneus longus (PER) and extensor digitorum longus (EDL) muscles. ROIs were eroded by one voxel to minimize partial volume effects at muscle interfaces. Dixon images were aligned to DT‐MRI data using elastix (v4.9, https://elastix.lumc.nl), and the resulting transformations were applied per ROI. The mean, median and standard deviation of conventional DT‐MRI metrics, fractional anisotropy (FA) and mean diffusivity (MD), were then calculated per ROI. To reduce noise and fat bias, we excluded voxels with signal‐to‐noise ratios (SNRs) < 20^9^ or fat fraction (FF) > 80%.

#### Random permeable barrier model analyses

The RPBM considers water diffusion to be hindered by a network of randomly oriented, semi‐permeable membranes, resembling the cross‐sectional topology of skeletal muscle.^11^ Accordingly, the median radial diffusivity of each muscle ROI, representing diffusion in the cross‐section of muscle fibres, was fitted across all three diffusion times using the RPBM (*Data S1*), to estimate the characteristic fibre diameter, *a*, and myofibre membrane permeability, κ. Pixel‐wise maps were also generated for examining intra‐muscle, pixel‐wise variation in RPBM metrics.

### Tissue histology

Cross‐sectional biopsy cryosections (10 μm thick) were cut using a Leica CM3050 S cryostat (Leica Biosystems, Wetzlar, Germany). Slides were stored at −20°C prior to staining. After staining, sections were imaged using a ZEISS Axio Scan.Z1 fluorescent microscope (Carl Zeiss Microscopy GmbH, Jena, Germany) with a ×10 objective and images were processed and stitched using ZEN Blue (v3.4; Carl Zeiss Microscopy).

#### Tissue visualization

Before haematoxylin and eosin (H&E) staining, slides were equilibrated to room temperature (RT) for 30 min. They were then fixed in ice‐cold acetone for 5 min, air‐dried at RT for 30 min and rinsed in deionized water. Slides were stained in haematoxylin for 3 min and briefly rinsed in deionized water, and the staining was developed in running tap water for 5 min. Destaining was performed by dipping slides into acid ethanol 8–12 times, after which they were rinsed in tap water and then deionized water. Finally, slides were stained for 30–45 s in eosin. H&E‐stained sections were then dehydrated and mounted in Pertex (Histolab, Los Angeles, CA, USA).

#### Myofibre size analysis

Prior to laminin staining, slides were equilibrated to RT and then blocked for 30 min with 5% milk powder (FrieslandCampina, Amersfoort, the Netherlands) in phosphate‐buffered saline containing 0.05% Tween (PBST). Cryosections were then stained with rabbit anti‐laminin (1:1000, L9393, Sigma‐Aldrich) for 2 h at RT. Following washing with PBST, the secondary antibody goat anti‐rabbit‐conjugated Alexa Fluor® 750 (1:1000, A21039, Thermo Fisher Scientific) was incubated for an hour at RT. Lastly, after washing again with PBST, cryosections were mounted with ProLong™ Gold antifade reagent (P36930, Thermo Fisher Scientific) and stored at 4°C before imaging. Laminin images were automatically quantified using a set of Fiji macros (v1.51),^21^ as previously described.^22^ The minimum Feret diameter, perimeter, circularity and cross‐sectional area (CSA) were obtained per myofibre for selected samples, and measurements in pixels were converted to micrometres. Circularity and CSA measurements were used for quality control (*Data S1*).

#### Fibrosis analysis

Slides were kept at RT for 30 min, fixed in 4% paraformaldehyde for 10 min, fixed in 100% ethanol for 5 min and air‐dried at RT for 30 min, after which they were rinsed in deionized water. Thereafter, cryosections were stained with Sirius‐red solution (MilliporeSigma, Burlington, MA, USA) for 45 min, washed twice with 0.5% acetic acid water for 5 min, rinsed with deionized water, dehydrated and mounted in Pertex. Images were background‐corrected using Adobe Photoshop 2021 (Adobe Inc., San Jose, CA, USA), and spots of dirt, blood vessels and perimysium were erased. Sirius‐red‐positive areas were quantified using Fiji and normalized to the total sample area. Two observers assessed the images and the mean of their results was used for analysis. Where these results differed by more than 5%, the images were visually assessed by both observers, who together selected the more representative of the two.

### Statistical analysis

We used R (v3.5, R Foundation for Statistical Computing, Vienna, Austria) for all statistical analyses. One‐ and two‐way analyses of variance (ANOVAs) were used to assess intra‐group (inter‐muscle) differences and inter‐group differences in muscle ROI measures, respectively. Post hoc comparisons were performed with Tukey's range test if data demonstrated equal variances or the Games–Howell test if they did not, with correction for multiple comparisons. Inter‐individual variability in histology (TA only) and RPBM parameters (all muscles) was assessed using an *F*‐test for normally distributed data and the Fligner–Killeen (FK) test for non‐normal data. Associations between RPBM parameters and histology, between RPBM and functional measures, and diffusion parameters and age were evaluated via standardized linear regression. For these analyses, we used RPBM metrics from the TA only. We considered *P*‐values < 0.05 to be statistically significant.

## Results

Demographic data for participants who underwent MRI are shown in *Table* *1*, along with clinical data for BMD patients.

**Table 1:** Participant characteristics for Becker muscular dystrophy (BMD) patients and healthy controls included in the magnetic resonance imaging component of this study

	BMD (*n* = 13)	Controls (*n* = 9)	*P*‐value
Age, years [range]	41 [20–59]	44 [23–65]	0.63
Height, m	1.79 (0.08)	—	—
Weight, kg	81.4 (16.2)	84.8 (13.9)	0.6
Body mass index, kg/m^2^	25.5 (5.5)	—	—
Ambulatory, *n* (%)	12 (92%)	—	—
6MWT, m	529 (213)	—	—
NSAA	27 [19]	—	—
Rise‐from‐floor time, s	4.53 (2.64)	—	—
10‐m run/walk velocity, m/s	2.09 (1.30)	—	—
Whole‐thigh fat fraction (3 slices), %	18.4 [48.8]	—	—

*Note*: Data are expressed as mean (standard deviation) or median [interquartile range], except where noted.

Abbreviations: 6MWT, 6‐min walk test; NSAA, North Star ambulatory assessment.

### Diffusion‐tensor magnetic resonance imaging

**Figure 2:**
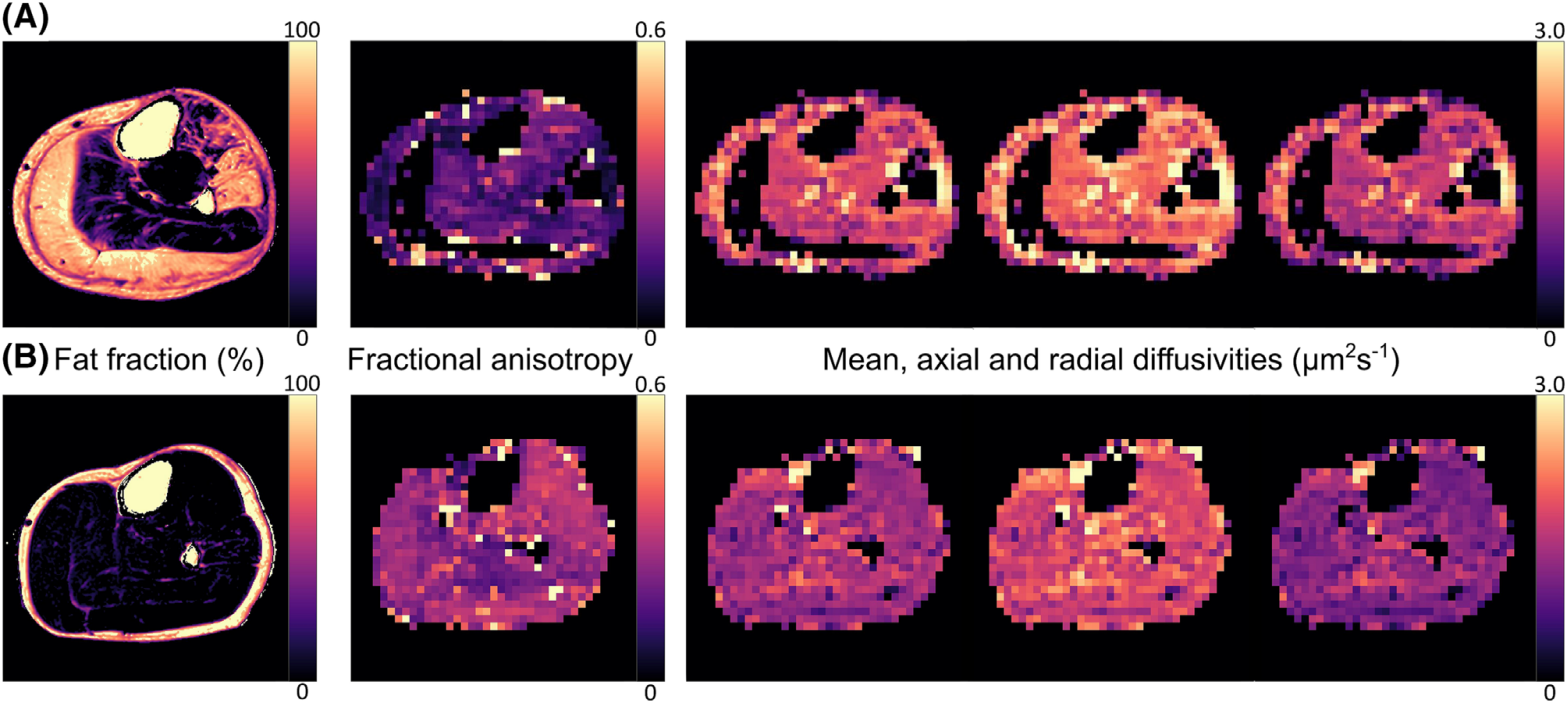
Representative lower leg ‘Dixon’ chemical‐shift‐based water‐fat separation images and stimulated‐echo diffusion‐tensor magnetic resonance imaging (STE‐DT‐MRI) parameter maps from a 59‐year‐old Becker muscular dystrophy (BMD) patient (A) and a 58‐year‐old male healthy control (B). STE‐DT‐MRI data were acquired with a diffusion time of 330 ms. The BMD patient shows severe fat replacement in the gastrocnemius medialis and lateralis and in the peroneus longus and extensor digitorum longus muscles. This leads to signal voids in these regions in the DT‐MRI data, which were acquired with comprehensive fat suppression.^20^

Representative FF and DT‐MRI parameter maps are shown in *Figure* *2*. No data were excluded due to artefacts or poor compliance. Fat‐, SNR‐ and outlier‐related exclusions are listed in the Supporting Information, *Data S2*.

#### Conventional diffusion‐tensor magnetic resonance imaging parameters vary with diffusion time and differ between muscles

**Figure 3:**
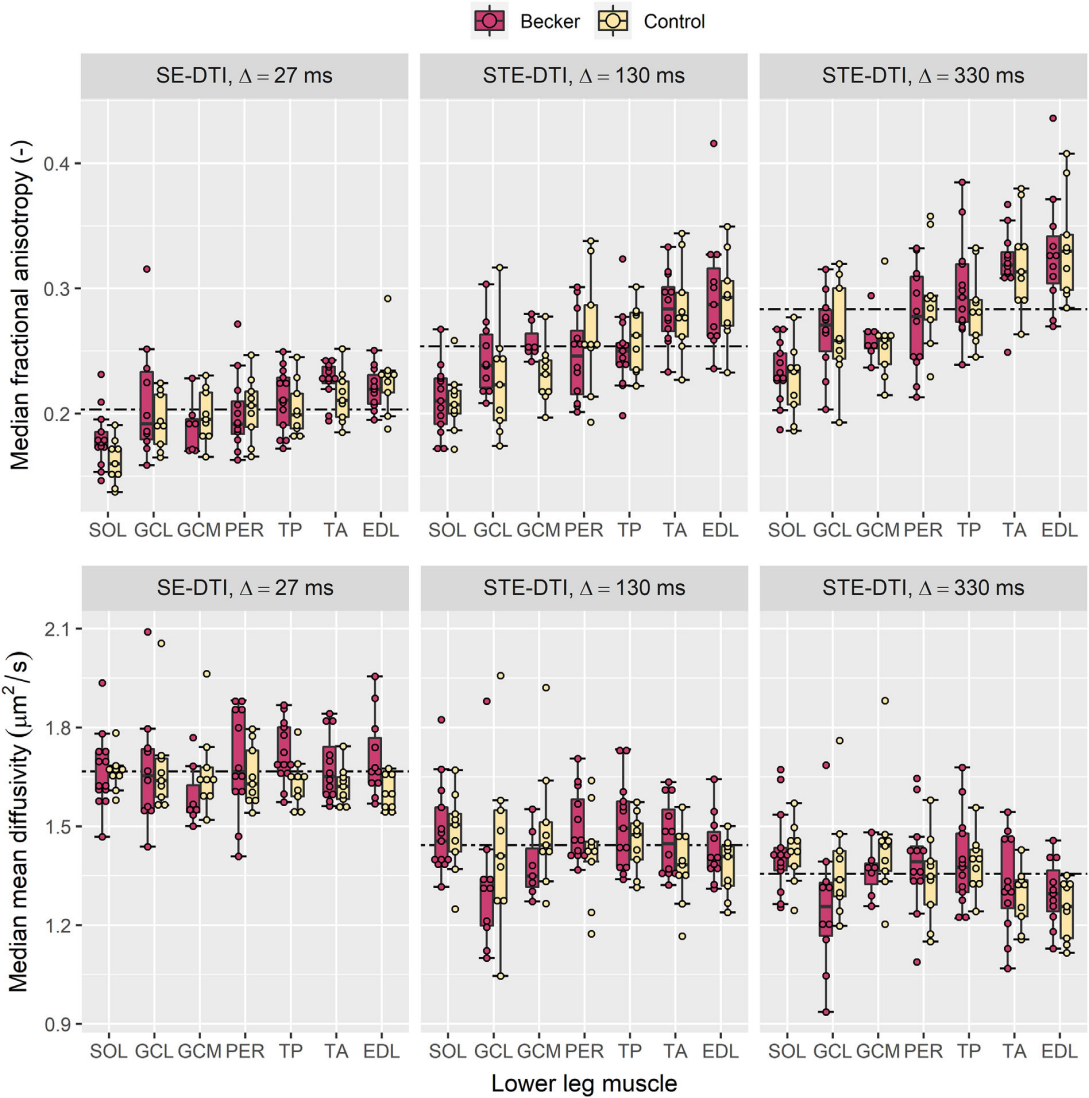
Boxplots showing fractional anisotropies (FA, top row) and mean diffusivities (MD, bottom row) derived from spin‐echo and stimulated‐echo diffusion‐tensor imaging (SE‐DTI and STE‐DTI, respectively) at three diffusion times, Δ, increasing from left to right. Median region‐of‐interest (ROI) measures are shown for Becker muscular dystrophy (BMD) patients and healthy controls over seven muscles of the lower leg: soleus (SOL), lateral gastrocnemius (GCL), medial gastrocnemius (GCM), peroneus longus (PER), tibialis posterior (TP), tibialis anterior (TA) and extensor digitorum longus (EDL). The boxplots represent median values by thick lines, with hinges corresponding to the 25th and 75th percentiles and dots approximating the raw data points. Boxplots are also ordered by median FA, highlighting a pattern of FA differences across the lower leg where the SOL has the lowest FA and the EDL has the highest, at all diffusion times. Dashed lines represent averages of all ROIs across all participants per diffusion time, showing that FA increases with diffusion time, whereas MD decreases. No statistically significant differences were observed between BMD patients and controls.

Boxplots of median FA and MD per ROI for both groups of participants are shown in *Figure* *3*. The mean FA over all muscles and subjects increased with increasing diffusion time, whereas MD decreased. There were no differences in FA between groups at any diffusion time: for Δ = 27 ms, *F*(1, 133) = 1.59, *P* = 0.21; for Δ = 130 ms, *F*(1, 133) = 0.18, *P* = 0.67; and for Δ = 330 ms, *F*(1, 133) = 0.05, *P* = 0.83. This was also true of MD: for Δ = 27 ms, *F*(1, 133) = 3.45, *P* = 0.06; for Δ = 130 ms, *F*(1, 133) = 0.41, *P* = 0.52; and for Δ = 330 ms, *F*(1, 133) = 0.05, *P* = 0.82. Inter‐muscle differences and age associations in conventional DT‐MRI parameters are shown in *Data* *S2*.

#### Random permeable barrier model parameters

**Figure 4:**
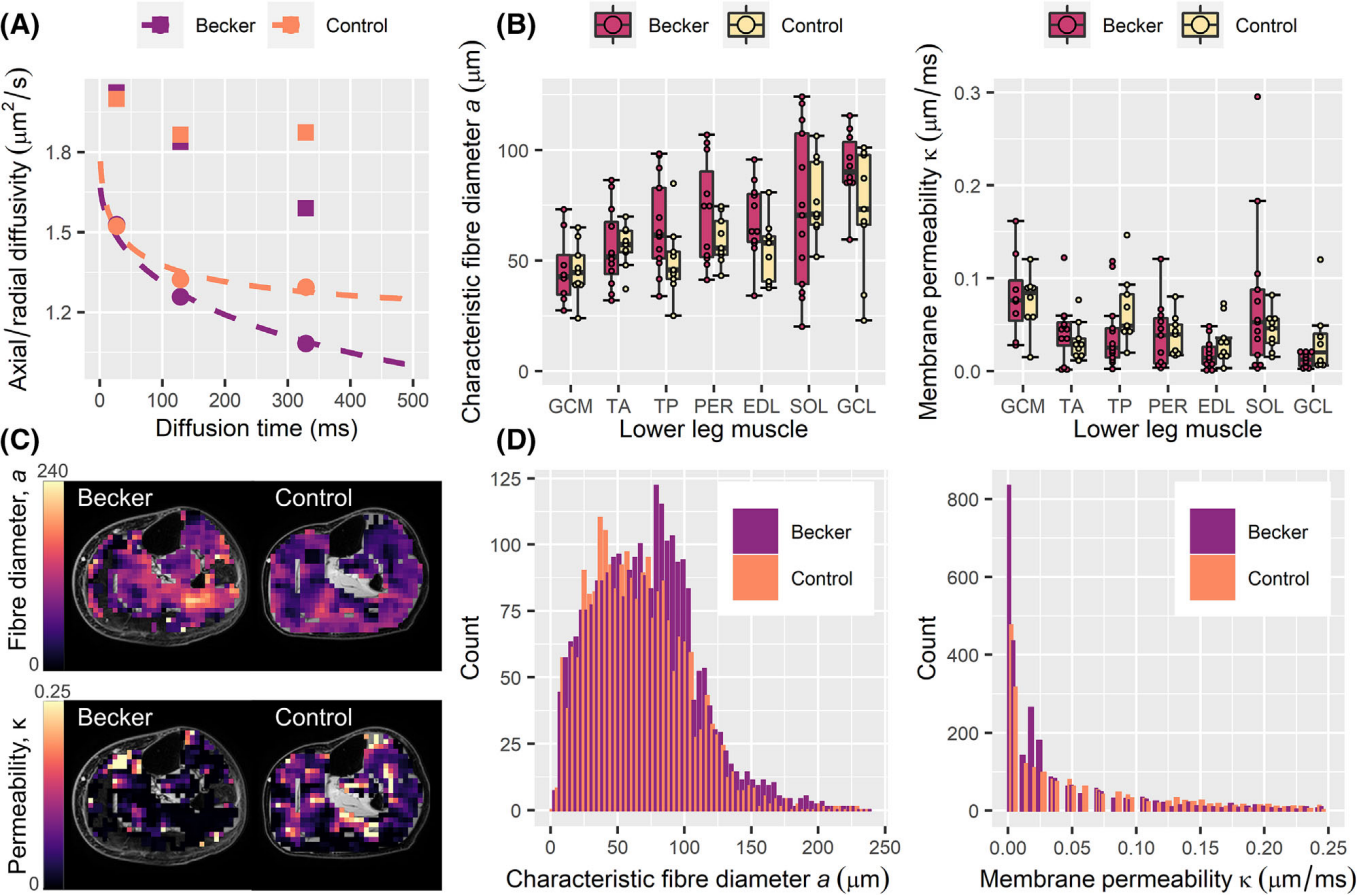
Characteristic fibre diameter and membrane permeability results obtained from the random permeable barrier model (RPBM) in Becker muscular dystrophy (BMD) patients and healthy controls. (A) Scatter plots showing the time dependence of axial and radial diffusivities in the soleus muscles of a 58‐year‐old control and a 59‐year‐old BMD patient. Squares and circles denote axial and radial diffusivities, respectively, per diffusion time, and dashed lines indicate fits of the RPBM to the radial diffusivity data per participant. (B) Boxplots showing *a* and κ for each group over the medial gastrocnemius (GCM), tibialis anterior (TA), tibialis posterior (TP), peroneus longus (PER), extensor digitorum longus (EDL), soleus (SOL) and lateral gastrocnemius (GCL) muscles. Median values are represented by thick lines, with hinges corresponding to the 25th and 75th percentiles, and boxplots are ordered by median fibre diameter. Two‐way analysis of variance showed globally larger fibre diameters in BMD patients versus controls, but no per‐muscle differences between groups. (C) Parameter maps, overlaid on matching ‘Dixon’ chemical‐shift‐based water‐fat separation water images, showing *a* and κ at the mid‐calf level for the aforementioned BMD patient and control. (D) Histograms showing pixel‐wise distributions of *a* and κ over the TA muscles of all participants, where BMD patients tend to show larger fibre diameters.

The RPBM was applied to estimate muscle fibre sizes and membrane permeabilities. Representative RPBM fits, group boxplots, and parameter maps and distributions are shown in *Figure* *4*. ROI‐wise fibre diameters ranged from 9.3 to 124.2 μm over all participants, whereas membrane permeability ranged from 0.001 to 0.3 μm/ms.

#### Random permeable barrier model fibre diameters are larger in Becker muscular dystrophy patients, but membrane permeabilities do not differ

Fibre diameter was globally larger in BMD patients than controls—*F*(1, 133) = 5.63, *P* = 0.019—with mean (SD) = 68.0 (25.3) μm versus 59.4 (19.2) μm, respectively. The assumption of homogeneity of variance was violated (*P* = 0.003); therefore, the Huber–White heteroscedasticity correction was used. Post hoc analyses showed no statistically significant differences in fibre diameter between patients and controls in any individual muscles. Membrane permeability did not differ between groups—*F*(1, 133) = 1.28, *P* = 0.261.

#### Random permeable barrier model fibre diameter variability within a muscle is similar between groups

The inter‐group, intra‐muscle variance in fibre diameter, given by the interquartile range (IQR) of pixel‐wise RPBM parameters in the TA muscle per subject, was similar between groups: mean (SD) = 56.5 (14.5) μm in BMD patients versus 55.9 (11.6) μm in controls, *P* = 0.918. The membrane permeability variance was also similar between patients and controls: median [IQR] = 0.126 [0.074] versus 0.101 [0.043] μm/ms, respectively, with *P* = 0.862.

#### Random permeable barrier model parameters differ between muscles in Becker muscular dystrophy patients and healthy controls

RPBM parameters were compared between muscles to explore known inter‐muscle differences in BMD disease involvement.^1^ Significant differences in fibre diameter were observed in BMD patients—*F*(6, 71) = 3.88, *P* = 0.002—and controls—*F*(6, 56) = 4.32, *P* = 0.001 (*Figure* *4*). Post hoc comparisons showed fibre diameter differences in certain muscles: In patients, the GCL showed larger fibre diameters than the EDL (*P* = 0.045), the GCM (*P* < 0.001), the TA (*P* = 0.001) and the TP (*P* = 0.034); and in controls, the SOL showed larger fibre diameters than the GCM (*P* = 0.008) and the TP (*P* = 0.044). Membrane permeability differed between muscles in patients—*F*(6, 71) = 3.37, *P* = 0.006—but not in controls—*F*(6, 56) = 1.98, *P* = 0.084. Post hoc comparisons highlighted lower permeabilities in the GCL versus SOL (*P* = 0.025) and GCL versus GCM (*P* = 0.029) in patients. No clear proximo‐distal RPBM parameter trends were observed within muscles in either group.

### Tissue histology

**Figure 5:**
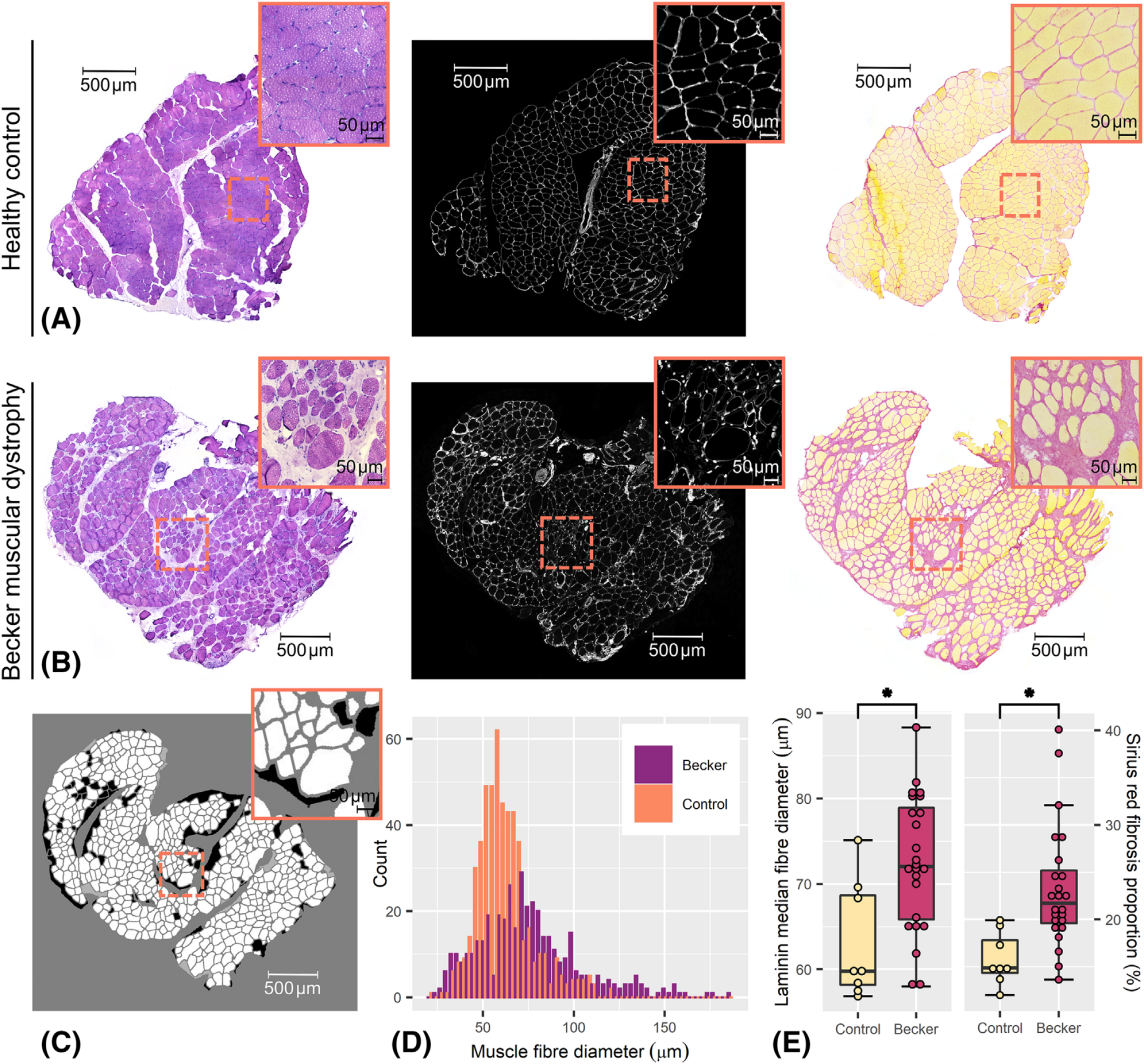
Representative histology images from this study, with zoomed‐in region inset, and examples of intra‐ and inter‐individual distributions. (A) From left to right, example haematoxylin and eosin, laminin and Sirius‐red images, respectively, from a tibialis anterior muscle sample taken from a 48‐year‐old healthy control; and (B) from a 37‐year‐old Becker muscular dystrophy (BMD) patient. (C) An example of semi‐automatic segmentation of the BMD laminin image shown in (B). (D) Histograms showing the distributions of muscle fibre diameters obtained by segmentation of laminin images in (A) and (B); it can be seen that the BMD laminin image contains a broader distribution of fibre sizes, with a trend towards larger fibres, as compared with the control. (E) Boxplots showing the inter‐individual distributions of median laminin‐derived fibre size (left) and the relative area of fibrosis (right), per sample. BMD patients show significantly larger fibre sizes, along with a greater proportion of fibrosis, as compared with controls; however, the inter‐individual variance in median fibre diameter was similar between patients and controls. *Statistically significant group difference at the *P* = 0.05 level.

Only samples that passed quality control for all three stainings were selected for further analyses. Exclusions are detailed in *Data* *S2*. See *Figure* *5* for representative images.

#### Laminin segmentation shows larger, more variable fibre sizes in Becker muscular dystrophy patients

Group and individual fibre diameter distributions are illustrated in *Figure* *5* and summarized in *Table* *2* along with other fibre size and shape metrics. Median values were used for group comparisons as distributions in individuals were skewed. On a group level, BMD patients again showed larger muscle fibre diameters than controls, with a mean (SD) = 72.5 (7.9) versus 63.2 (6.9) μm, *P* = 0.006; however, the inter‐individual variance in fibre diameter did not differ between groups—with *F*(23, 7) = 0.65, *P* = 0.746—and fibre circularity was similar (*P* = 0.222).

**Table 2:** Histology results from the tibialis anterior, including donor age, muscle fibre size and shape metrics from laminin segmentations, and fibrosis proportions derived from Sirius‐red stains

	BMD (*n* = 24)	Controls (*n* = 8)	*P*‐value
Age, years [range]	41 [19–66]	40 [23–48]	0.76
Laminin fibre segmentation			
Cross‐sectional area, μm^2^	6009 [2207]	3903.9 [1428.8]	0.008*
Minimum Feret diameter, μm	72.5 (7.9)	63.2 (6.9)	0.006*
Perimeter, μm	321.3 [61.8]	258.3 [49.7]	0.010*
Circularity	0.75 [0.03]	0.76 (0.02)	0.222
Sirius‐red fibrosis analysis			
Fibrosis proportion, %	21.7 [5.6]	14.9 [3.4]	<0.001*

*Note*: Data are expressed as mean (standard deviation) or median [interquartile range].

Abbreviation: BMD, Becker muscular dystrophy.

* Statistically significant at the *P* = 0.05 level.

In contrast to the RPBM results, intra‐individual variance in fibre diameter, given by the IQR of fibre diameters per sample, was greater in BMD patients than controls: mean variance (SD) = 34.2 (7.9) versus 21.4 (6.9) μm, *P* ≪ 0.001.

#### Sirius‐red measures of fibrosis show larger fibrotic areas in Becker muscular dystrophy patients

Sirius‐red‐derived fibrosis areas are shown in *Figure* *5* and *Table* *2*. Of 32 fibrosis estimates, 2 deviated more than 5% between observers, who then selected the most representative estimate. BMD patients showed larger fibrotic areas than controls: median (IQR) = 21.7 (5.6)% versus 14.9 (3.4)%, *P* < 0.001. The inter‐individual variance in fibrotic area did not differ between groups, with FK χ^2^ = 2.57, *P* = 0.109.

### Comparisons between random permeable barrier model parameters and histology

We used linear regression to test the validity of RPBM‐derived fibre diameters and membrane permeabilities from the whole TA muscle as compared with fibre diameters and fibrotic areas from histology, respectively. Although the RPBM and laminin segmentation showed comparable mean fibre diameters, as described in the Diffusion‐tensor magnetic resonance imaging and Tissue histology sections and illustrated in *Figures* *4* and *5*, no association was observed in the five patients with good‐quality DT‐MRI and muscle biopsy data (*P* = 0.379). Neither was there an association between fibre diameter variances from the two techniques (*P* = 0.278). We did, however, observe an association between the fibrosis area and membrane permeability from the RPBM, with β = 0.93, *P* = 0.021.

## Discussion

In this study, we explored non‐invasive muscle‐fibre‐size and permeability parameters from the RPBM in BMD and verified these results against detailed quantitative histological measures of muscle fibre morphology and fibrotic areas. We showed that BMD patients have larger muscle fibres than healthy controls overall, and RPBM‐measured fibre diameters covered a similar range to those estimated via laminin segmentation of muscle biopsies. RPBM measures of membrane permeability were found to be similar between BMD patients and controls, contrary to expectation; however, membrane permeability was positively associated with fibrosis areas measured using Sirius‐red staining. These findings suggest that RPBM DT‐MRI can non‐invasively measure muscle changes associated with pathology development in BMD.

### Muscle fibre diameter and membrane permeability from the random permeable barrier model

Our RPBM‐derived fibre diameters, made over a large muscle volume, are larger in BMD patients than in controls and show similar distributions to laminin‐segmentation‐derived fibre diameters from the TA muscle, albeit with a larger range, as shown in *Figures* *4* and *5*. The RPBM has been shown to underestimate fibre diameters in vivo, as it approximates muscle microstructure as a square lattice. We accounted for this via a correction factor determined in silico,^23^ giving values consistent with our own histology data, as well as literature data from BMD^3^, ^24^ and controls.^25^ When comparing the RPBM and histology in patients who underwent DT‐MRI and muscle biopsy, however, the fibre diameter measures were not associated. This is likely due to lack of colocalization between the DT‐MRI and muscle biopsies. Further, the triceps surae muscles—the soleus and the gastrocnemius lateralis and medialis—did not show consistent trends towards higher fibre diameters in patients versus controls, despite being preferentially affected early in BMD.^26^

In terms of the inter‐individual variability of muscle fibre diameters, we observed no differences between BMD patients and controls with the RPBM or histology. However, histology showed differences in intra‐individual fibre diameter variability between groups, whereas the RPBM did not. This discrepancy may be due to the inherently lower SNR of pixel‐wise parameter estimates, which leads to bias and greater variability, or because large MRI volumes capture the broad variation of fibre size in across whole muscles,^24^ unlike local biopsy samples—perhaps masking smaller regional differences. This can be explored further using DT‐MRI and histology data from the same muscle location.

Our RPBM membrane permeability estimates did not differ between patients and controls, appearing to contradict past findings that sarcolemmal membrane permeability is increased in muscular dystrophies: due to local membrane disruptions known as ‘delta lesions’^27^ and damage from mechanical stress.^15^ In this study, our RPBM implementation may have had limited sensitivity to permeability differences due to the limited range of diffusion times used (27–330 ms). At shorter diffusion times, water molecules are largely confined to the interior of the myofibre, whereas at longer diffusion times, they come into greater contact with the sarcolemmal membrane.^13^ An expanded RPBM DT‐MRI protocol with longer diffusion times may provide better specificity for detecting sarcolemmal permeability changes in BMD. Nevertheless, despite the lack of differences in membrane permeability between BMD patients and controls in our study, we did observe a strong, positive association between RPBM membrane permeability and Sirius‐red‐measured fibrosis area, which suggests sensitivity of RPBM permeability to disease progression. We should, however, consider the possible influence of fibrosis itself on diffusion parameters, as the endomysium is not explicitly modelled in the RPBM. A simulation study by Berry et al. has shown that—out of fibrosis, fibre size, membrane permeability and oedema—fibrosis had the least effect on the diffusion signal,^28^ though even a small effect could be enough to mask the relatively minor changes in permeability seen in BMD. The possible relationship between RPBM measures and fibrosis could be explored further via independent MRI‐based fibrosis measures, such as a recently demonstrated contrast‐enhanced T_1_ mapping approach that shows marked changes in BMD.^29^ Alternatively, further applications in DMD, where membrane leakiness is expected to be greater than in BMD, or in Marfan syndrome, where membrane permeability is normal, but substantial interstitial fibrosis can be found, may provide further insights on the value of RPBM metrics.

### Conventional diffusion‐tensor magnetic resonance imaging parameters

In addition to our RPBM metrics, DT‐MRI produces ‘conventional’ metrics reflecting the directionality of diffusion—whether it is restricted to a particular direction (FA)—and the magnitude of diffusion, independent of direction (MD). These metrics have been linked to skeletal muscle pathophysiology, including fibre atrophy,^30^ oedema^31^ and denervation.^32^ Few studies have applied DT‐MRI to muscular dystrophies,^8^, ^9^, ^10^, ^33^, ^34^, ^35^, ^36^ and most have examined small patient cohorts. Previous work by our group in DMD showed significant, though inconsistent FA and MD differences between patients and controls,^9^ and an earlier application of spin‐echo DT‐MRI in our BMD cohort demonstrated no FA or MD differences.^8^ Even so, our novel application of stimulated‐echo DT‐MRI here, which uses longer diffusion times to probe larger length scales, showed no between‐group FA or MD differences. We did, however, observe more inter‐muscle FA differences at longer diffusion times, which may stem from fibre‐type differences,^37^ and inter‐muscle MD differences only appeared at long diffusion times. Diffusion times from 90 to 250 ms are thought to provide optimal contrast between healthy and injured muscles.^23^ Our diffusion times of 130 and 330 ms fall in and around this range, suggesting that our data are sensitive to muscle pathological changes. Indeed, McDowell et al. demonstrated clear FA and MD differences in DMD using stimulated‐echo DT‐MRI.^10^ That differences are observed between DMD patients and controls and not the BMD patients and controls in our study could be attributed to BMD's heterogeneity, as it demonstrates highly variable presentation and progression.^38^

### Histological measures of muscle fibre size and fibrosis

Fibre‐size variability and endomysial fibrosis in BMD have been documented in several studies, dating back to the 1970s.^3^, ^5^, ^39^, ^40^ Relative areas of muscle fibre and fibrotic tissue from histology have recently been used as endpoints in a phase II clinical trial in BMD^5^; however, muscle fibre size itself has not yet been applied as an endpoint. Recent quantitative work by Ripolone et al. showed lower relative muscle fibre areas, larger fibrotic areas and greater variability in biceps brachii fibre CSA in BMD patients as compared with controls.^40^ In agreement with these findings, our histology data showed greater intra‐individual variance in muscle fibre size in BMD patients as compared with controls, as well as larger relative fibrosis areas. The increased variability in fibre size in BMD has been attributed to atrophy and hypertrophy of muscle fibres, both of which come about due to repeated cycles of muscle damage and regeneration.^3^

In our histology data, we showed a larger median muscle fibre diameter in BMD patients versus controls, suggesting significant fibre hypertrophy in this BMD cohort. Ripolone et al., however, observed no inter‐group differences in mean CSA, which was measured manually on myosin‐ATPase‐stained sections.^40^ When comparing mean CSAs, as per Ripolone et al., we continued to observe between‐group differences; therefore, the lack of agreement between our studies could instead be due to between‐muscle differences—as we obtained biopsies from the TA rather than the biceps brachii—or differences in segmentation approaches. We used automated segmentation, which allowed us to analyse whole muscle samples as opposed to manually selected microscope fields. This may have served to better capture the full diversity of fibre sizes seen in BMD.

### Limitations

In this study, relatively few patients who underwent MRI also had muscle biopsies; in those that did, biopsies were obtained from the contralateral leg. Whereas BMD disease progression is considered to occur symmetrically,^1^ it is unclear whether this is true of microstructural changes. This potential asymmetry, along with the small number of matched samples, may explain the lack of association in this study between RPBM‐ and histology‐derived muscle fibre sizes. In future, these parameters should be compared using biopsy samples colocalized to the DT‐MRI. We also acquired a limited number of diffusion times, which increases the likelihood of the RPBM fit being deflected by noise; however, both participant groups showed comparable SNRs at longer diffusion times, meaning this effect is expected to be similar between patients and controls.

## Conclusions

DT‐MRI data allow the use of the RPBM to non‐invasively estimate muscle fibre diameters. In this study, we show larger and more variable muscle fibre diameters in BMD versus healthy controls. Given that RPBM metrics can quantify changes in muscle fibre size that are associated with regeneration, without the need for muscle biopsies, they show particular promise as imaging biomarkers for muscular dystrophies. Future work should examine whether changes in RPBM fibre size over time can predict the replacement of muscle tissue with fat and fibrosis in these diseases.

## Conflict of interest statement

D.C., T.A.D., L.G.M.H., N.M.V., Z.K., T.T.J.V., M.T.H., S.A., S.G.D., J.J.G.M.V., M.P., V.R. and P.S. report no relevant disclosures. A.A.R. discloses being employed by LUMC, which has patents on exon skipping technology, some of which have been licensed to BioMarin and subsequently sublicensed to Sarepta. As co‐inventor of some of these patents, A.A.R. is entitled to a share of royalties. A.A.R. further discloses being ad hoc consultant for PTC Therapeutics, Sarepta Therapeutics, Regenxbio, Alpha Anomeric, BioMarin Pharmaceuticals Inc., Eisai, Entrada, Takeda, Splicesense, Galapagos and AstraZeneca, with past ad hoc consulting for CRISPR Therapeutics, Summit PLC, Audentes Santhera, Bridge Bio, Global Guidepoint and GLG Consultancy, Grünenthal, Wave and BioClinica. A.A.R. also reports having been a member of the Duchenne Network Steering Committee (BioMarin), being a member of the scientific advisory boards of Eisai, Hybridize Therapeutics, Silence Therapeutics and Sarepta Therapeutics, and being a former member of the scientific advisory boards of ProQR and Philae Pharmaceuticals. LUMC also received speaker honoraria from PTC Therapeutics and BioMarin Pharmaceuticals and funding for contract research from Italfarmaco, Sapreme, Eisai, Galapagos, Synaffix and Alpha Anomeric. A.A.R. received project funding from Sarepta Therapeutics. E.H.N. reports ad hoc consultancies for Wave, Santhera, Regenxbio and PTC, and he worked as investigator of clinical trials of Italfarmaco, NS Pharma, Reveragen, Roche, Wave and Sarepta outside the submitted work. H.E.K. reports research support from Philips Healthcare during the conduct of the study, consultancy for PTC Therapeutics and EspeRare and trial support from ImagingDMD‐UF outside the submitted work. All reimbursements for A.A.R., E.H.N. and H.E.K. were received by the LUMC. No personal financial benefits were received.

## Supporting information


**Data S1.** Methods

**Data S2.** Results
