# Supplementary material for: Diffusion‐tensor magnetic resonance imaging captures increased skeletal muscle fibre diameters in Becker muscular dystrophy
Source: J Cachexia Sarcopenia Muscle. 2023 May 1;14(3):1546–57. doi: 10.1002/jcsm.13242 (PMC10235880; doi:10.1002/jcsm.13242)
Supplement: Supplementary file 1 — Data S1. Methods [file JCSM-14-1546-s002.docx]

**Diffusion-tensor MRI captures increased skeletal muscle fibre diameters in Becker muscular dystrophy**

**JOURNAL OF CACHEXIA, SARCOPENIA AND MUSCLE**

Donnie Cameron^1^*, Tooba Abbassi-Daloii^2^, Laura G.M. Heezen^2^, Nienke M. van de Velde^3,4^, Zaïda Koeks^3^, Thom T.J. Veeger^1^, Melissa T. Hooijmans^5^, Salma el Abdellaoui^2^, Sjoerd G. van Duinen^6^, Jan J.G.M. Verschuuren^3,4^, Maaike van Putten^2,4^, Annemieke Aartsma-Rus^2,4^, Vered Raz^2^, Pietro Spitali^2,4^, Erik H. Niks^3,4^, Hermien E. Kan^1,4^*

1. C.J. Gorter MRI Center, Department of Radiology, Leiden University Medical Center, Leiden, The Netherlands

2. Department of Human Genetics, Leiden University Medical Center, Leiden, The Netherlands

3. Department of Neurology, Leiden University Medical Center, Leiden, The Netherlands

4. Duchenne Center Netherlands

5. Radiology and Nuclear Medicine, Amsterdam University Medical Center, University of Amsterdam, Amsterdam Movement Sciences, Amsterdam, The Netherlands
6. Department of Pathology, Leiden University Medical Center, Leiden, The Netherlands

***Correspondence:** Donnie Cameron, C.J. Gorter MRI Center , Department of Radiology-C3Q, Leiden University Medical Center, Albinusdreef 2, 2333 ZA Leiden, The Netherlands. Tel.: +31 71 526 3501; E-mail: [D.Cameron@lumc.nl](mailto:H.E.Kan@lumc.nl)

Hermien E. Kan, C.J. Gorter MRI Center , Department of Radiology-C3Q, Leiden University Medical Center, Albinusdreef 2, 2333 ZA Leiden, The Netherlands. Tel.: +31 71 526 6097; E-mail: [H.E.Kan@lumc.nl](mailto:H.E.Kan@lumc.nl)

**SUPPORTING INFORMATION, METHODS S1**

**Magnetic resonance imaging localisation and sequence parameters**

All imaging stacks were oriented perpendicular to the tibia and centred around the thickest part of the lower leg. The Dixon 2D gradient-recalled echo sequence was played after localisers with: TR = 210 ms; 3 echoes, with TE_1_ = 4.4 ms and ∆TE = 0.8 ms ; flip angle = 8°; field-of-view = 180 mm × 180 mm; matrix size = 180 × 180; acquired in-plane resolution = 1 mm × 1 mm, reconstructed to 0.47 mm × 0.47 mm; 23 slices with 10 mm thickness and a 5 mm gap; and 2 signal averages.

Spin-echo and stimulated-echo DT-MRI echo-planar-imaging sequences were applied with: TR/TE = 5,000/58 ms; field-of-view = 384 mm × 384 mm; matrix size = 96 × 96; in-plane resolution = 4 mm × 4 mm; 9 slices with 6 mm thickness and 3 mm gap; *b*-values = 0 and 400 s/mm^2^; 12 diffusion directions; and a sensitivity-encoding factor of 1.7 in the phase-encoding (anterior–posterior) direction. Both DT-MRI sequences used comprehensive fat suppression comprising spectral attenuated inversion recovery, slice-select-gradient reversal, and Dixon olefinic fat suppression (DOFS).[1] To satisfy the requirements for DOFS, we acquired DT-MRI data at six different TE values, corresponding to water-fat phase offsets of 30°, 90°, 150°, 210°, 270°, and 330°. Further to these DOFS acquisitions, conventional spectrally-fat-suppressed spin-echo DT-MRI was applied using similar parameters, with the following differences: TR/TE = 2,990/50 ms; field-of-view = 192 mm × 192 mm; in-plane resolution = 2 mm × 2 mm; *b*-values = 0 and 450 s/mm^2^; 15 diffusion directions; and six signal averages. These data also been published elsewhere.[1, 2] Spin-echo DT-MRI sequences had a diffusion gradient duration, δ, of 12 ms and a diffusion time, Δ, of 27 ms, while stimulated-echo DT-MRI had δ = 5 ms and Δ values of 130 ms and 330 ms, corresponding to the 100 and 300 ms TMs, respectively.

Dixon and DT-MRI stacks were planned with overlapping slice locations to permit accurate region-of-interest (ROI) delineation. Finally, to determine the DT-MRI signal-to-noise ratio (SNR), 16 noise scans were acquired with RF excitation switched off, allowing noise to be estimated on a per-voxel basis. The total duration of the scan was approximately one hour, including a short break between the upper-leg protocol (not shown) and the lower-leg protocol.

*Diffusion tensor MRI processing pipeline.* The data processing pipeline, outlined in Fig. 1 in the main text, consisted of the following steps:

1. Denoising via an overcomplete local principal component analysis filter;[3]
2. Registration of all DOFS DT-MRI echo-shifts to the first echo of the non-weighted data in elastix (v4.9, <https://elastix.lumc.nl>) using 3D affine transformations;
3. Phase-unwrapping using a magnitude-sorted, multi-clustering method;[4]
4. DT-MRI Dixon fat-water decomposition,[5] yielding fat and water images, B_0_ maps, and R_2_* maps;
5. Susceptibility distortion correction using DT-MRI-derived B_0_ maps;[6]
6. Correction of diffusion *b*-matrices for registration-related rotations using rotation matrices from the affine transformations;[7]
7. Correction of diffusion *b*-values for cross-terms arising from STE-DT-MRI imaging gradients;[8, 9]
8. DT-MRI parameter calculation using the UCL Camino Diffusion MRI Toolkit (University College London, UK; downloaded 2019-07-11) with weighted linear-least-squares regression;[10] and
9. SNR map determination for *b* = 0 and 400 data by dividing these by the standard deviation of the noise estimated from the 16 noisy image volumes.

Throughout all steps, data were visually assessed for artefacts and issues such as water-fat swap, residual phase errors, or misregistrations.

*Random permeable barrier model fitting*

Radial diffusivity (RD), representing diffusion across the short axes of muscle fibres, was calculated as the average of the second and third diffusion eigenvalues. Median RDs were fitted using a two-parameter nonlinear-least-squares approach ([github.com/NYU-DiffusionMRI/RPBM](https://github.com/NYU-DiffusionMRI/RPBM)). The fibre diameter, *a,* was calculated as 6.29 divided by the surface-to-volume ratio.[11] Free diffusivity, *D_0_*, was fixed to the average of the primary diffusion eigenvalues, or axial diffusivities (ADs) representing diffusion along the long axis of muscle fibres, obtained at Δ = 130 and 330 ms.[12, 13]

**Tissue histology quality control**

*Myofibre size analysis.* Small regions of endomysium that were erroneously identified as muscle fibres and large, longitudinal, or partly-longitudinal, fibres were excluded based on separate histogram filters for BMD patients and controls: apparent fibres smaller than the second percentile and larger than the 95^th^ percentile were removed. Whole samples were excluded when insufficient fibres remained after filtering (fewer than 100) or when substantial freezing and cutting damage was observed.

**REFERENCES**

1. Burakiewicz J, Hooijmans MT, Webb AG, Verschuuren JJGM, Niks EH, Kan HE. Improved olefinic fat suppression in skeletal muscle DTI using a magnitude-based Dixon method. Magnetic Resonance in Medicine. 2018;79(1):152-9.

2. Hooijmans MT, Froeling M, Koeks Z, Verschuuren JJGM, Webb A, Niks EH, et al. Multi-parametric MR in Becker muscular dystrophy patients. NMR in Biomedicine. 2020;33(11):e4385.

3. Manjón JV, Coupé P, Concha L, Buades A, Collins DL, Robles M. Diffusion Weighted Image Denoising Using Overcomplete Local PCA. PLOS ONE. 2013;8(9):1-12.

4. Maier F, Fuentes D, Weinberg JS, Hazle JD, Stafford RJ. Robust phase unwrapping for MR temperature imaging using a magnitude-sorted list, multi-clustering algorithm. Magnetic Resonance in Medicine. 2019;73(4):1662-8.

5. Reeder SB, Wen Z, Yu H, Pineda AR, Gold GE, Markl M, et al. Multicoil Dixon chemical species separation with an iterative least-squares estimation method. Magnetic Resonance in Medicine. 2004;51(1):35-45.

6. Jezzard P, Balaban RS. Correction for geometric distortion in echo planar images from B0 field variations. Magnetic Resonance in Medicine. 1995;34(1):65-73.

7. Leemans A, Jones DK. The B-matrix must be rotated when correcting for subject motion in DTI data. Magnetic Resonance in Medicine. 2009;61(6):1336-49.

8. Mattiello J, Basser PJ, Le Bihan D. The b matrix in diffusion tensor echo-planar imaging. Magnetic Resonance in Medicine. 1997;37(2):292-300.

9. Güllmar D, Haueisen J, Reichenbach JR. Analysis of b-value calculations in diffusion weighted and diffusion tensor imaging. Concepts in Magnetic Resonance Part A. 2005;25(1):53-66.

10. Jones DK, Basser PJ. 'Squashing peanuts and smashing pumpkins': How noise distorts diffusion-weighted MR data. Magnetic Resonance in Medicine. 2004;52(5):979-93.

11. Berry DB, Englund EK, Galinsky V, Frank LR, Ward SR. Varying diffusion time to discriminate between simulated skeletal muscle injury models using stimulated echo diffusion tensor imaging. Magnetic Resonance in Medicine. 2021;85(5):2524-36.

12. Sigmund EE, Novikov DS, Sui D, Ukpebor O, Baete S, Babb JS, et al. Time-dependent diffusion in skeletal muscle with the random permeable barrier model (RPBM): Application to normal controls and chronic exertional compartment syndrome patients. NMR in Biomedicine. 2014;27(5):519-28.

13. Fieremans E, Lemberskiy G, Veraart J, Sigmund EE, Gyftopoulos S, Novikov DS. In vivo measurement of membrane permeability and myofiber size in human muscle using time-dependent diffusion tensor imaging and the random permeable barrier model. NMR in Biomedicine. 2017;30(3):e3612-e.
