# Supplementary material for: Diffusion‐tensor magnetic resonance imaging captures increased skeletal muscle fibre diameters in Becker muscular dystrophy
Source: J Cachexia Sarcopenia Muscle. 2023 May 1;14(3):1546–57. doi: 10.1002/jcsm.13242 (PMC10235880; doi:10.1002/jcsm.13242)
Supplement: Supplementary file 2 — Data S2. Results [file JCSM-14-1546-s001.docx]

**Diffusion-tensor MRI captures increased skeletal muscle fibre diameters in Becker muscular dystrophy**

**JOURNAL OF CACHEXIA, SARCOPENIA AND MUSCLE**

Donnie Cameron^1^*, Tooba Abbassi-Daloii^2^, Laura G.M. Heezen^2^, Nienke M. van de Velde^3,4^, Zaïda Koeks^3^, Thom T.J. Veeger^1^, Melissa T. Hooijmans^5^, Salma el Abdellaoui^2^, Sjoerd G. van Duinen^6^, Jan J.G.M. Verschuuren^3,4^, Maaike van Putten^2,4^, Annemieke Aartsma-Rus^2,4^, Vered Raz^2^, Pietro Spitali^2,4^, Erik H. Niks^3,4^, Hermien E. Kan^1,4^*

1. C.J. Gorter MRI Center, Department of Radiology, Leiden University Medical Center, Leiden, The Netherlands

2. Department of Human Genetics, Leiden University Medical Center, Leiden, The Netherlands

3. Department of Neurology, Leiden University Medical Center, Leiden, The Netherlands

4. Duchenne Center Netherlands

5. Radiology and Nuclear Medicine, Amsterdam University Medical Center, University of Amsterdam, Amsterdam Movement Sciences, Amsterdam, The Netherlands
6. Department of Pathology, Leiden University Medical Center, Leiden, The Netherlands

***Correspondence:** Donnie Cameron, C.J. Gorter MRI Center , Department of Radiology-C3Q, Leiden University Medical Center, Albinusdreef 2, 2333 ZA Leiden, The Netherlands. Tel.: +31 71 526 3501; E-mail: [D.Cameron@lumc.nl](mailto:H.E.Kan@lumc.nl)

Hermien E. Kan, C.J. Gorter MRI Center , Department of Radiology-C3Q, Leiden University Medical Center, Albinusdreef 2, 2333 ZA Leiden, The Netherlands. Tel.: +31 71 526 6097; E-mail: [H.E.Kan@lumc.nl](mailto:H.E.Kan@lumc.nl)

**SUPPORTING INFORMATION, RESULTS S2**

**Diffusion-tensor MRI quality control**

We drew 154 muscle regions of interest (ROIs) for the entire study cohort and applied these to diffusion-tensor MRI (DT-MRI) data at 3 diffusion times, producing 462 ROIs overall. Of these, 39 were excluded because no voxels remained after erosion and thresholding by signal-to-noise ratio (SNR) and fat fraction. The SNR of the diffusion data was higher in controls at a short diffusion time, Δ = 27 ms, with a mean (SD) SNR of 83.5 (9.5) versus 78.3 (11.3) in Becker muscular dystrophy (BMD) patients (*p* = 0.023). At longer diffusion times, however, SNRs were similar between groups: 27 for both groups at Δ = 130 ms and 22 for both groups at Δ = 330 ms.

Supplemental Fig. S1 shows examples of pixel-wise distributions of conventional DT-MRI parameters: fractional anisotropy (FA), mean diffusivity (MD), axial diffusivity (AD), and radial diffusivity (RD). Distributions were seen to be skewed, and median values were therefore used for further analyses.


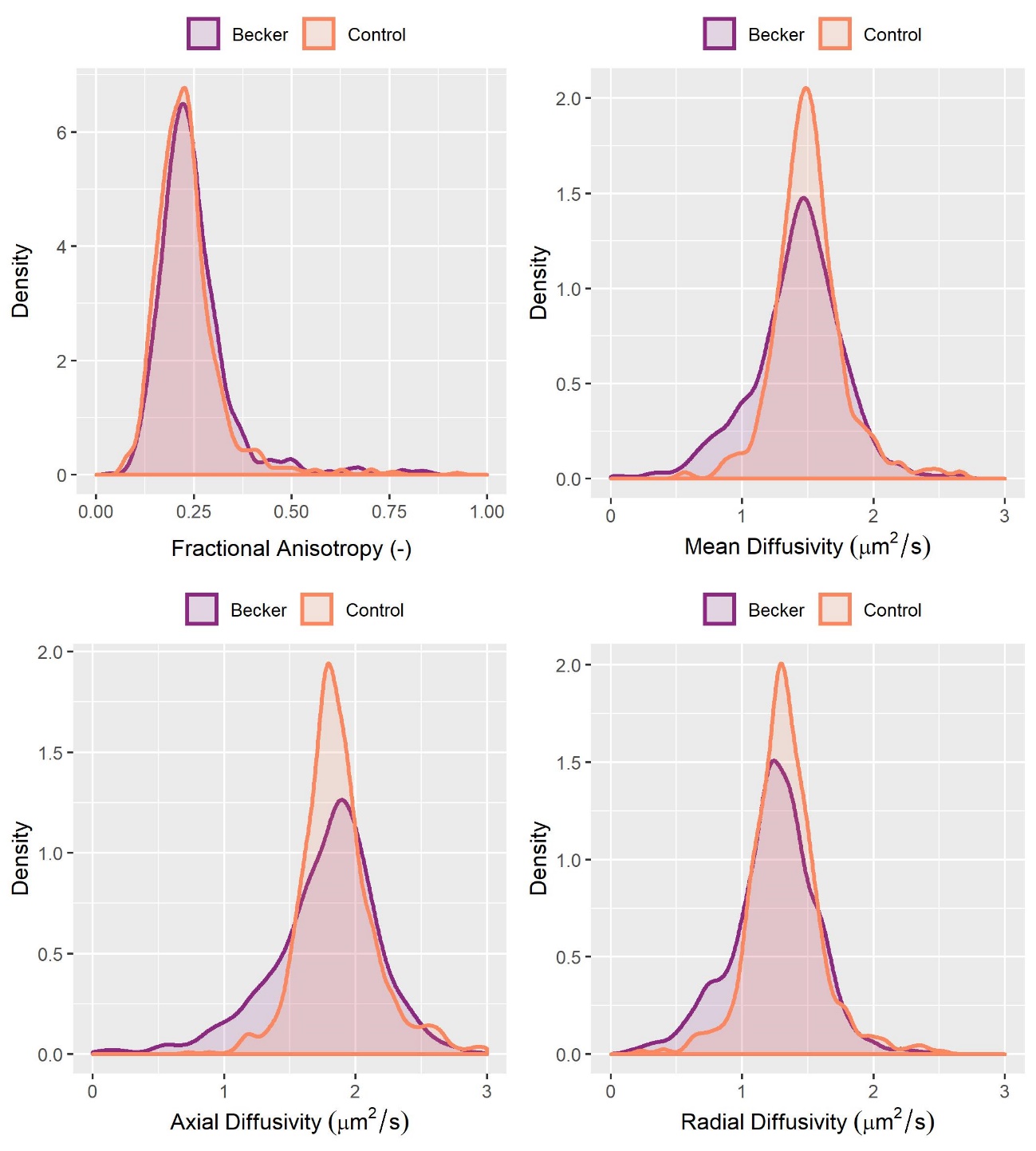


**Supplemental Figure S1** *Density plots showing the distributions of conventional diffusion tensor imaging (DT-MRI) parameters in the soleus muscles of a 58-year-old healthy control and a 59-year-old Becker muscular dystrophy (BMD) patient.. The total area under each curve integrates to one. Note the skew towards lower diffusivities in the BMD patient*

For the random permeable barrier model (RPBM) results, out of 144 ROIs, we excluded three extreme outliers from three different patients: two ROIs with excessively high κ (GCM – 0.83 and PER – 0.43 μm/ms) and correspondingly low *a* (5.9 and 5.97 μm); and a third, GCL ROI with high *a* (209.3 μm) and low κ (0.001 μm/ms).

*Conventional DT-MRI parameters differ between muscles in BMD patients and healthy controls*. Overall, muscles of the posterior compartment tended to have lower median FA values than those of the anterior compartment. One-way analysis of variance (ANOVA) in each participant group confirmed this, showing statistically-significant differences in FA between muscles in BMD and healthy volunteer groups at all diffusion times (*p* < 0.001 in all cases), and in MD for both groups at longer diffusion times. Post-hoc Tukey’s range tests were also performed. These showed multiple between-muscle differences, with greater numbers of differences at longer diffusion times: in BMD patients, the number of between-muscle differences increased from 2 at Δ = 27 ms, to 6 at Δ = 130 ms, to 9 at Δ = 330 ms; in healthy controls, the number of between-muscle differences started at 6 at Δ = 27 ms, increased to 8 at Δ = 130 ms, and remained at 8 at Δ = 330 ms.

*Conventional DT-MRI parameters are associated with age in BMD patients and healthy controls.* In the whole cohort, median FA was negatively associated with age at Δ = 130 ms alone (β = −0.26, *p* = 0.002, adjusted *R*^2^ = 0.06). This held after subsetting by group (β = −0.26, *p* = 0.02, adjusted *R*^2^ = 0.06 in patients; and β = −0.27, *p* = 0.033, adjusted *R*^2^ = 0.06 in controls). Median MD was positively associated with age at Δ = 130 ms (β = 0.25, *p* = 0.002, adjusted *R*^2^ = 0.06), and this held for patients and controls (β = 0.28, *p* = 0.011, adjusted *R*^2^ = 0.07; and β = 0.25, *p* = 0.047, adjusted *R*^2^ = 0.05, respectively). In controls, MD was also associated with age at Δ = 330 ms (β = 0.32, *p* = 0.01, adjusted *R*^2^ = 0.09).

**Tissue histology quality control**

Out of all 38 BMD samples, ten were excluded due to poor-quality laminin results, one due to poor Sirius red, and four due to poor-quality laminin and Sirius red stainings. Out of all control samples, two were excluded due to poor laminin alone, one due to poor Sirius red, and eight due to poor laminin and Sirius red stainings. After quality-based exclusions, and preferential removal of duplicate samples from the earlier, 2011 natural history study, 24 BMD samples remained for further analysis, along with 8 control samples. Of those who underwent DT-MRI, this left five with matched RPBM and biopsy metrics.
